# Experimental and Clinical Evaluation of Capsular and Parenchymal Total Liver Perfusion

**DOI:** 10.1155/1992/53863

**Published:** 1992

**Authors:** Katerina Kotzampassi, Efthimios Eleftheriadis, Homeros Aletras

**Affiliations:** 1 Department of Surgery University of Thessaloniki Thessaloniki Greece; 2 33, Them. Sofouli str Thessaloniki GR-546 55 Greece

## Abstract

Liver blood flow measurements obtained from both the liver surface and deep within the parenchyma, were correlated in an effort to assess the usefulness of laser-Doppler flowmetry for non-invasive monitoring of total liver blood flow, the probe being positioned on either the surface or within the liver parenchyma.

In 23 Wistar rats and 10 biliary surgery patients, anaesthetized prior to gallbladder removal, liver microcirculation was measured at 4 points on the capsular surface, and consequently at 4 points deep within the parenchyma, using probes connected to a laser-Doppler flowmeter. The findings revealed that laser-Doppler measurements on the liver surface and within the parenchyma were well correlated, as no statistically significant differences were found either in rats or humans. It is concluded that laser- Doppler flowmetry for monitoring of total liver perfusion can be applied either on the capsular surface or within the hepatic parenchyma.


*HPB Surgery*, 1992, Vol. 6, pp. 99–104
Reprints available directly from the publisher
Photocopying permitted by license only

© Harwood Academic Publishers GmbH
Printed in the United States of America

### EXPERIMENTAL AND CLINICAL EVALUATION OF CAPSULAR AND PARENCHYMAL TOTAL LIVER PERFUSION

### Liver Microcirculation

KATERINA KOTZAMPASSI, EFTHIMIOS ELEFTHERIADIS and HOMEROS ALETRAS

*Department of Surgery, University of Thessaloniki, Thessaloniki, Greece*

*(Received 27 January 1992)*

Liver blood flow measurements obtained from both the liver surface and deep within the parenchyma, were correlated in an effort to assess the usefulness of laser-Doppler flowmetry for non-invasive monitoring of total liver blood flow, the probe being positioned on either the surface or within the liver parenchyma.

In 23 Wistar rats and 10 biliary surgery patients, anaesthetized prior to gallbladder removal, liver microcirculation was measured at 4 points on the capsular surface, and consequently at 4 points deep within the parenchyma, using probes connected to a laser-Doppler flowmeter. The findings revealed that laser-Doppler measurements on the liver surface and within the parenchyma were well correlated, as no statistically significant differences were found either in rats or humans. It is concluded that laser-Doppler flowmetry for monitoring of total liver perfusion can be applied either on the capsular surface or within the hepatic parenchyma.

KEY WORDS: Liver microcirculation, laser-Doppler flowmetry

Evaluation of liver blood flow is fundamental to describing hepatic physiology and biochemistry and understanding the pathophysiological states of this organ. The recent progress in both liver transplantation[1,2] and ischemic treatment of hepatic tumors[3], has reawakened our interest in blood flow in the liver.

A variety of techniques have been employed throughout the years in attempts to measure the hepatic blood flow in experimental animals and in humans[4,5,6]. These methods, the majority of which are invasive, led to a picture of a homogenous perfusion throughout the depth of hepatic tissue in any given region of the normal liver. However, recent data, has given rise to some debate on the distribution of blood flow within the parenchyma and the liver surface[7].

The aim of the present study is the estimation of hepatic perfusion and the correlation of measurements obtained at different regions on the surface and within the liver parenchyma, experimentally in the rat and in clinical practice, in man. The method used for this assessment is the newly developed laser-Doppler technique.

Address correspondence to: E. Eleftheriadis, M.D., 33, Them. Sofouli str, GR-546 55 Thessaloniki, Greece

### MATERIAL AND METHOD

### *Laser-Doppler Technique*

The laser-Doppler flowmeter employed in the study was the Periflux PF2B, (Perimed, Sweden). The operating principle of this instrument has been described in detail previously[8]. The probes used were the self-adhesive single fibre probe (PF319: 2L) for liver surface measurements and the needle probe (PF302) for measurements within the parenchyma. The self-adhesive single fibre probe is a new tool added to the armentarium of laser-Doppler probes[9]. It is constituted of one optical fibre with an overall diameter of 0.5 mm, with a small latex sheet attached to its angular tip. This latex sheet adheres well to moist surfaces, and keeps the probe in position without glues or other mechanisms, thus permitting a very stable laser-Doppler signal to be obtained. All measurements were performed with a signal processing Periflux filter at 4 kHz and time constant of the output amplifier at 0.2 sec. The laser-Doppler flowmetry readings in arbitrary units of flux were continuously transferred and stored in a serially connected IBM-PS2 PC, by the use of the Perisoft® software (Perimed, Sweden), for further analysis.

### *Animal Studies*

Twenty-three male Wistar rats (450–500 gr) were included in the study. Rats were deprived of food but allowed free access to water for an 18 hour period before the experiment. After light ether anaesthesia, through a middle-line laparotomy, a 22G polyethylene tube (Angiocath®) was inserted into the caudal aorta, for continuous monitoring of the arterial pressure via a transducer (S10/S11, Gaeltec, Ltd) connected to a linear pen recorder (Multitrace2, Lectromed, Sweden).

### *Hepatic Microcirculation*

The laser-Doppler self-adhesive probe was attached to the liver surface, achieving optical coupling, which was identified by the green light signal on the flowmeter. Blood flow was measured for a 30 sec period at 4 random sites on the main hepatic lobe. After these measurements were performed, the laser-Doppler needle probe was introduced through the lumen of a 22G intravenous cannula, inserted into 4 different points of the liver parenchyma and blood flow was assessed for a 30 sec period at each site.

### *Clinical Studies*

Ten biliary surgery male patients aged 45–56 yrs (median 48 yr) were included in the study, after informed consent had been obtained. All patients had no history of jaundice, cholangitis or hepatic function disturbance. After right subcostal incision the right hepatic lobe was exposed and, under arterial pressure monitoring, both surface and parenchymal measurements were performed as previously described, using the same probes and before any surgical manipulation.

### *Statistical Analysis*

All data analysis are expressed as the mean ± SEM. One-way analysis of variance was conducted using the one factor ANOVA (Stat-View, BrainPower Inc, CA) for repeated measurements for correlation of the findings within and between the subjects. Dunnet *t*-test and Fisher's test were applied to determine differences among groups if a significant F value was obtained. Probability values $<0.05$ were considered significant.

### RESULTS

The mean ± SEM of laser-Doppler flowmetry in arbitrary units of flux both from the surface and parenchyma are presented in Figure 1 for rats and Figure 2 for humans. The one-way ANOVA revealed an F value of 0.853, $p = 0.7144$ for rats and 1.625, $p = 0.0654$ for humans, that is there is no significant differences among flux values within and between measurements as well as between capsular and parenchymal measurements.

### DISCUSSION

The metabolic functions of the liver are influenced by impairment of the hepatic circulation. It is, therefore, clinically important to be able to monitor liver blood flow both under physiological and pathophysiological conditions. Orthotopic liver transplantation has become widely accepted for hepatic disease treatment[1,2], but its early postoperative management remains difficult because of the relatively complex interactions between the many causes of early liver graft dysfunction, such as

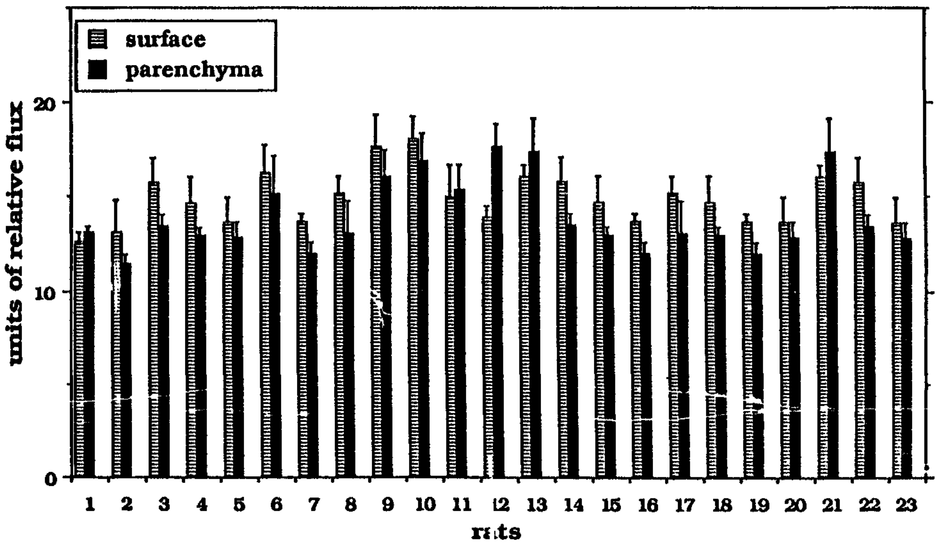

**Figure 1** Histograms of laser-Doppler flowmetry measurements in 23 rats. Bars represent the mean ± SEM value of 4 measurements performed either on the surface (open bars) or within the parenchyma (black bars).

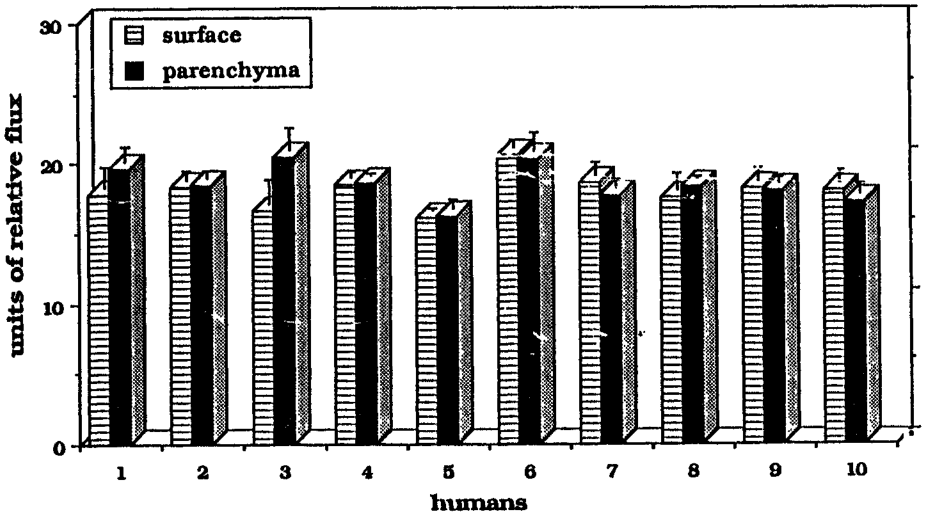

**Figure 2** Histograms of laser-Doppler flowmetry measurements in 10 humans. Bars represent the mean ± SEM value of 4 measurements performed either on the surface (open bars) or within the parenchyma (black bars).

ischemic damage, hepatic artery thrombosis or acute early rejection. Ischemic treatment of hepatic tumors by the use of implantable occluders applied to the hepatic artery for repeated temporary hepatic dearterialization[3] would be facilitated by the estimation of liver and/or tumor regional blood flow, as flow monitoring might provide an early warning sign as well as an index of the efficacy of the therapy.

For all these purposes non-invasive monitoring of hepatic blood flow is essential. The laser-Doppler technique used in this study is a newly developed method for microvascular blood flow assessment in tissues. The measurable volume of a laser-Doppler flowmeter constitutes a hemisphere with a radius of about 1 mm[10] which is assumed to be constant for a given probe's geometry in most tissues[11]. Additionally, the laser-Doppler velocimetry exhibits a high sensitivity to changes in blood flow, compatible to values obtained with other invasive methods[12,13], and responds with high reproducibility, so it may be used for continuous monitoring of rapid flow changes. The linearity of laser-Doppler output with total liver blood flow over the range 0 to 2 ml/min/g was found to have an r value greater than 0.97, equal to that of $^{85}Kr$ washout curves in the same tissue[10].

However, little data is available concerning possibilities and, perhaps, limitations of using the method for estimating liver blood flow[7,12,14]. One of these studies, conducted by Ardvinsson[7] on six pigs, after portal vein or hepatic artery occlusion, raised some doubts about the homogenous perfusion throughout the depth of the hepatic parenchyma. It is obvious, however, that these differences are probably due to the vessels' occlusion and there is no correlation with the unmanipulated liver we studied. The findings of the present investigation conducted in rats and humans, revealed that blood flow on the liver surface is well correlated with the blood flow within the liver parenchyma. Additionally, no statistically significant variations were observed in measurements within an individual subject or between subjects under similar conditions, both in rats and humans.

Our findings of homogenous blood perfusion throughout the liver supports the conclusions of others, based on studies using $^{14}C$-labelled antipyrine[15] and radioactive microspheres[16]. Furthermore, anatomical studies using non-diffusible indicators such as red blood cells and serum-albumin[17,18] do not suggest any compartmentalization of flow within the liver substance, so the suggestion[7] that the measurable volume of liver blood flow at a random probe position is too small to reliably reflect changes of total blood flow remains of no practical interest with respect to the intact liver. The homogenous perfusion throughout the entire depth of the liver — as documented by our performing repeated measurements which exhibited a small within measurements variation — makes the performance of a single-point measurement somewhere within the liver an accurate estimation of total liver blood flow. On the other hand the insertion of the laser probe into the liver parenchyma does not constitute a special problem, regarding the accuracy of readings, as the needle is so fine it does not affect the physiological architecture of the tissue.

Previous studies have confirmed that the laser-Doppler probe applied to the liver surface is more sensitive to the changes of blood flow through the arterial than the portal system[12]. Anatomical studies, in which it was demonstrated that terminal arteries were lying between the two layers of the liver capsule, can partially explain these findings[19]. On the other hand, Leiberman *et al.*[20] obtained close agreement between results derived from beta emission recordings of $^{85}Kr$ clearance and electromagnetically measured total liver blood flow, thus demonstrating an overall accuracy of $^{85}Kr$ clearance recordings. As this type of $^{85}Kr$ detection allows an assessment of events only in superficial liver tissue, it was inferred from these studies that perfusion near the surface of the liver mirrors flow in the deeper circulation, thereby giving additional support to the concept of intralobar flow homogeneity. Thus, our findings, too, provide evidence that liver blood flow may be equally accurately measured within the parenchyma or on the surface.

The real need of a continuous monitoring of liver graft perfusion, led Payen *et al.*[21] to use implantable pulsed Doppler microprobes on the hepatic artery and portal vein in 10 patients undergoing orthotopic liver transplantation, for one week postoperatively. Although, the use of the laser-Doppler self-adhesive fibre probe is a simpler method of continuous monitoring, some technical problems remain, such as how the fiber probe could be externalized through the abdominal wall and removed at the end of the monitoring period.

In summary, laser-Doppler flowmetry with needle or self adhesive probes is a useful method for rapid measurement and monitoring of total liver blood flow and can be applied either on the capsular surface or within the hepatic parenchyma, according to the specific need and conditions.

### *References*

1. Iwatsuki, S., Starzl, T., Toda, S. *et al.* (1988) Liver transplantation in the treatment of bleeding oesophageal varices. *Surgery*, **104**, 697
2. Bird, G.L.A., O'Grady, J.G., Harvey, F.A.H., Calne, R.Y. and Williams, R. (1990) Liver transplantation in patients with alcoholic cirrhosis. Selection criteria and rates in survival and relapse. *Br. Med. J. (Clin. Res.)*, **301**, 15

3. Ahren, B. and Bengmark, S. (1988) Reviewing article: Ischemic and metabolic treatment of heptic tumors. *HPB Surgery*, **1**, 3
4. Johnson, D.J., Muhlbacher, F., Wilmore, D.W. (1985) Measurement of hepatic blood flow. *J. Surg. Res.*, **39**, 470
5. Mathie, R.T. (1986) Hepatic blood flow measurement in inert gas clearance. *J. Surg. Res.*, **41**, 92
6. Gouma, D.J., Coelho, C.U., Schlegel, J., Fisher, J.D., Li, Y.F. and Moody, F.G. (1986) Estimation of the hepatic blood flow by hydrogen gas clearance. *Surgery*, **99**, 439
7. Arvidsson, D., Svensson, H. and Haglung, U. (1987) A comparison between laser-Doppler values obtained from the surface and the parenchyma of the liver. In: *Microcirculation—an update*, Tsuchiya M. *et al.* editors, Elsevier Science Publishers BV, Amsterdam, Vol 1, p. 376
8. Nilsson, G.E. (1990) Perimed's LDF flowmeter. In: *Laser Doppler flowmetry*, Shepherd, A.P., Öberg, P.A., editors, Kluwer Academic Publishers, Boston, p. 57
9. Salerud, E.G. and Öberg, P.A. (1987) Single-fibre laser Doppler flowmetry. A method for deep tissue perfusion measurements. *Med. & Biol. Eng. & Comput.*, **25**, 329
10. Almond, N.E. and Wheatley, A.M. (1991) Measurements of blood flow changes in the periphery of the liver using laser Doppler flowmetry. Proceedings 5th World Congress for microcirculation, Kentucky, abstr. 10
11. Kiel, J.W. and Shepherd, A.P. (1990) Gastrointestinal blood flow. In: *Laser Doppler flowmetry*, Shepherd, A.P., Öberg, P.A., editors, Kluwer Academic Publishers, Boston, p. 227
12. Arvidsson, D., Svensson, H. and Haglund, U. (1988) Laser-Doppler flowmetry for estimating liver blood flow. *Am. J. Physiol.*, **254**, G471
13. Richardson, P.D. and Withrington, P.G. (1981) Liver blood flow. I. Intrinsic and nervous control of liver blood flow. *Gastroenterol.*, **81**, 159
14. Shepherd, A.P., Riedel, G.L. and Ward, W.F. (1983) Laser Doppler measurements of blood flow within the intestinal wall and on the surface of the liver. In: *Microcirculation of the alimentary tract*, Koo, A., Lam, F.K., Swaje, L.M., editors, World Scientific, Singapore, p. 115
15. Darle, N. (1970) Xenon$^{133}$ clearance and liver blood flow. An experimental study in the cat. *Acta. Chir. Scand.*, **407** (Suppl.) 1
16. Greenway, C.V. and Oshiro, G. (1972) Intrahepatic distribution of portal and hepatic arterial blood flows in anaesthetized cats and dogs and the effects of portal occlusion, raised venous pressure and histamine. *J. Physiol*, **227**, 473
17. Field, C.D. and Andrews, W.H.H. (1968) Investigation of the hepatic arterial "space" under various conditions of flow in the isolated perfused dog liver. *Circ. Res.*, **23**, 611
18. Cohn, J.N. and Pinkerson, A.L. (1969) Intrahepatic distribution of hepatic arterial and portal venous flows in the dog. *Am. J. Physiol.*, **216**, 285
19. Glauser, F. (1953) Studies on intrahepatic arterial circulation. *Surgery*, **33**, 333
20. Leiberman, D.P., Mathie, R.T., Harper, A.M. and Blumgart, L.H. (1978) Measurement of liver blood flow in the dog using Krypton$^{85}$ clearance: a comparison with electromagnetic flowmeter measurements. *J. Surg. Res.*, **25**, 147
21. Payen, D.M., Fratacci, M.D., Dupuy, P., Gatecel, C., Vigouroux, C., Ozier, Y., Houssin, D. and Chapuis, Y. (1990) Portal and hepatic arterial blood flow measurements of human transplanted liver by implanted Doppler probes: Interest for early complications and nutrition. *Surgery*, **107**, 417

*(Accepted by S. Bengmark 5 May 1992)*

